# Teeth and Toothache

**Published:** 1893-04

**Authors:** 


ARTICLE VII.

### TEETH AND TOOTH-ACHE.

Of teeth it may be said that that man is happiest who is unconscious of them. We do not mean as an ornament. It is true that some people, as Montaigne says, take great care to black their teeth, and hate to see them white; whilst others paint them red." Europeans have a taste of their own in the matter, and love to see them white. For proof consult contemporary fiction *passim*, where even the villains have a fine set to show, and know it. Let Herrick sing of the rubies and pearls of his Julia's mouth; teeth for the present purpose are not jewels. They are to be regarded as the "*fons et origo mali*," and that evil is toothache.

Toothache is as old as sin, and as universal. To erring man it might figure as a form of final torture. It must have been part of the punishment of our primeval parents, whose doom we inherit. The first that an infant knows of teeth is pain; from the cradle to the grave they are an active source of annoyance. Some there be, indeed, who say not without pride that they never had a touch of toothache in their lives. But call not a man happy till he is dead. Hereafter writhing in anguish, they shall assuredly repent the premature boast. And there are strong men and the like who misuse their teeth to lift surprising weights, or, emulating that terror of the Spaniards and hero of the *Revenge* Sir Richard Grenville, chew glasses up without a grimace. "Blind mouths" (to pervert Milton's phrase), they do not look to the end—the fevered gums, the dull unceasing ache, the shooting spasm, as if a red-hot needle were thrust into the brain. If a man does altogether escape the fell disease, one is tempted to ascribe to him a low order of nervous organization. He must be "only an animal, only sensible in the duller parts" Nay, he is even lower than that, for animals, too, have toothache, and especially such as possess a high degree of intelligence. The dog and the horse are well-known sufferers. On the authority of a quaint old French book on the subject, we may add the wolf; and the hippopotamus "endures quite a great pain from its teeth, so that it is constrained to get out of the water to find a remedy."

This book is one of the earliest modern authorities on toothache that we have discovered. It is scientific, as science went in 1622. Its author, one Maistre Arnauld Gilles, was apparently court dentist for he dedicated his book to Marie Henriette de Bourbon, sister to the reigning king; and it is published at the appropriate sign of the Three Golden Teeth, in Paris. It is remarkable, we may say in passing, that the literature of toothache is so meagre. An ailment of such ancient standing in the world's history might be expected to obtain more frequent and detailed notice. Such modern treatises as exist are purely technical, and undeserving of the name of literature. There is in them nothing historical, nothing humane and sympathetic to the view of the sufferer. Even in the ordinary life of to-day there is no disease which gains us so little pity from our friends. It is not fatal, they say, and are apt to be impatient with our groans. And we ourselves, once the attack is over, straightway forget what manner of torture it was, and go unthinkingly about our daily business. Now, this is surely wrong. It may be true that toothache never killed anybody directly; but assuredly, if analogy goes for anything, it has been the cause of crime and death. Imagine an absolute monarch with an obstinate tooth. It would be a grim amusement to him, almost a necessity, to sign a death-warrant. There have been martyrs to toothache in another than the ordinary usage of the term.

But to return to Maistre Arnauld. The first thing to note is that he advises the specialization of dentistry. "It is very necessary that dentists should have no other vocation." He has known instances where patients have died from hæmorrhage because the ignorant drawer of teeth did not know how to stop the bleeding. The world, he says, by way of peroration may think the title "Drawer of teeth" strange, and perhaps despise it. But Maistre Arnauld glories in it as very useful to the public, "and does not do, like an infinity of others, who, coming to this town [of Paris], call themselves grand operators. He is happy to do his task well, to take the little fee that is given him, and is never ill-content." It is only lately that in England the Royal College of Surgeons recognized dentistry

3

as a special branch of medicine. Some twenty-five years ago their dental certificate was established. Before that, the craft was confined to tooth-drawing mainly, and had for its professors the local barber, blacksmith, or watchmaker. We are now beginning to see that unlicensed practitioners do a lot of mischief. The ancient Egyptians were before us in this field; for Herodotus tells us that no doctor in Egypt was permitted to practice any but his own peculiar branch, and some attended solely to diseases of the teeth. Proofs of their skill have been found in some mummies at Thebas whose teeth were stuffed with gold.

So much for the disease; but what of the cure? Maistre Arnauld gives several prescriptions, but they are commonplace compared with more ancient remedies. Here are two methods from Pliny: Put your hands behind your back; bite off a piece of wood from a tree which has been struck by lightning, and apply it to the ailing tooth. Or you may fumigate the tooth with the tooth of another of the same sex—how that is done we are not told—and bind the canine tooth of an unburied corpse to it. Habdarrahman on Egyptain medicine advises that the molar of a dead man—whether buried or not apparently does not matter—be hung over the groaning sufferer, and the pain will abate. Others, again, say: "Burn a wolf's head and keep the ashes. They are a great remedy." It is difficult to cap the piquancy of such cures; but Sir Thomas More has done it, and his prescription has the advantage of not requiring such inaccessible materials. "I have heard it taught me," he says in 1557, "for the toothache to go thrice about a churchyard and never think on a fox's tail." This reminds one in its malicious pleasantry of "Don't nail his ears to the pump"; for the suggestion of foxes' tails in connection with churchyards, though not very obvious to the common man, must always and inevitably recur to those who tried the cure.

The man in dental anguish sometimes curses with Burns "the venomed stang that shoots his tortured gums alang." Sometimes, on the other hand, he prays. St. Augustine in his *Confessions* relates how he once suffered from "*dolor dentium*" (toothache), apparently in an aggravated form, for he could not speak. Thereupon he wrote on wax a prayer to God for the other brethren to repeat; and as soon as all were on their knees the pain went. "But what a pain! he says—"never since my tender age had I experienced the like." Southey, in his *Life of John Wesley*, tells of that eminent preacher that when his own tooth ached he prayed, and the pain left him. Unfortunately, ordinary men do not seem to have such efficacious faith. When the excruciation begins they must bare it philosophically; and on Shakespeare's authority toothache finds out just the weak place in the philosopher's armor of patience. In the middle ages the devout who were racked with pain had a special patron to whom they could call for deliverance. St. Apollonia, a martyr under the Emperor Philip, among other cruel indignities had her teeth pulled out. In consequence, she became toothache's tutelary saint, as her emblems—one of which is "holding a tooth in pincers"—sufficiently testify. And there would seem to have been yet another martyr, St. Blaize, who took cognizance of the disease. He was honored in the little town of St. Blazey, in Cornwall, where candles offered upon his altar were supposed to be an infallible cure for toothache.

Perhaps something may be added on the subject of toothpicks. These are said to have been invented in Italy. Certainly they were in common use among the Romans. In Martial's Epigrams there are frequent references to the "*dentiscalpium*," sometimes reviling its abuse, sometimes praising its use. The particular form of tooth-pick which Martial preferred was a pointed strip of mastic-wood; but, in default of that, he recommends a quill. Singularly enough, the useful instrument was regarded as an innovation in Queen Elizabeth's time.

The Bastard, in *King John*, sneers at

> Your traveller—
> He and his toothpick at my worship's mess.

Travellers in France and Italy, it seems, brought toothpicks back, and used them ostentatiously; and all those who affected foreign fashions sedulously imitated them. Commonly a case of toothpicks made of wood was carried about by fine gentlemen. A more violent eccentricity of fashion is pointed at by Sir Thomas Overbury, who describes a courtier as walking in St. Paul's "with a picktooth in his hat, a cape cloak, and a long stocking." Apparently the "Johnny" of the present day, who is so unremitting in his use of the homely quill, has inherited the tooth-pick and his flourishing display of it from the coxcombs that thronged the court of the Virgin Queen.—*Dominion Dental Journal.*

